# Does the Fast Alcohol Screening Test Accurately Distinguish Between Harmful and Severely Dependent Tiers of Alcohol Misuse?

**DOI:** 10.1093/alcalc/agab015

**Published:** 2021-03-23

**Authors:** Bev John, Simon Newstead, Robert Heirene, Ray Hodgson, Gareth Roderique-Davies

**Affiliations:** Addictions Research Group, School of Psychology & Therapeutic Studies, University of South Wales, Pontypridd CF37 1DL, UK; Addictions Research Group, School of Psychology & Therapeutic Studies, University of South Wales, Pontypridd CF37 1DL, UK; Brain & Mind Centre, School of Psychology, Science Faculty, University of Sydney, Sydney, NSW 2050, Australia; Addictions Research Group, School of Psychology & Therapeutic Studies, University of South Wales, Pontypridd CF37 1DL, UK; Addictions Research Group, School of Psychology & Therapeutic Studies, University of South Wales, Pontypridd CF37 1DL, UK

## Abstract

**Aims:**

Primary aim: to determine the efficacy of FAST (the Fast Alcohol Screening Test) for detecting harmful and dependent levels of alcohol use. Secondary aim: to compare the performance of the FAST to two short forms of the Alcohol Use Disorder Identification Test (AUDIT): the AUDIT-C and AUDIT-3.

**Methods:**

Data from 3336 individuals in South Wales, compiled from full AUDIT datasets, were examined. AUROC analysis, alongside measures of sensitivity and specificity of the FAST, AUDIT-C and AUDIT-3 were utilized for the identification of harmful and dependent alcohol use.

**Results:**

The FAST demonstrated efficacy in the identification of harmful and dependent levels of alcohol use, with superior performance to both the AUDIT-C and AUDIT-3.

**Conclusion:**

The present paper demonstrates the potential of the FAST as a cost- and time-effective method for appropriate screening and signposting in the stepped care model utilized by many health care and treatment services. Further studies are needed to ensure validity, both within the general population and for specific services and populations.

## Teaser

**Short Summary**: The FAST (Fast Alcohol Screening Test) (a brief screen for alcohol consumption, dependence and harm) demonstrates efficacy in its ability to identify and distinguish between both harmful and severely dependent tiers of alcohol use, within a population which predominantly exhibits risk-related alcohol usage. The FAST exhibits superior performance, in this respect, to the AUDIT-C and AUDIT-3 (which measure consumption only), providing additional evidence to further highlight the importance of not solely relying on alcohol consumption to classify tiers of harm.

## INTRODUCTION

Time- and cost-efficient screening methods for alcohol-related problems have been adopted by a wide range of health and social service settings and have also been utilized as research outcome measures (Gómez *et al.*, 2005; Jones, 2011; Coulton *et al.*, 2012; Fitzgerald *et al.*, 2012). The Fast Alcohol Screening Test (FAST: Hodgson *et al.*, 2002) is one such measure that was developed for use within busy medical care settings, such as hospital Accident and Emergency (A&E) departments, which have a high rate of admission for individuals who misuse alcohol. The FAST is composed of just 4 questions from the 10-item Alcohol Use Disorder Identification Test (AUDIT: Saunders and Babor, 1993), which has long been regarded as the gold standard for screening and assessment of hazardous, harmful or dependent alcohol use (Mansfield *et al.*, 2019). The AUDIT has been shown to perform with a high level of accuracy when measuring alcohol consumption risk, across gender, age and cultures (Saunders, Aasland, Amundsen, *et al.*, 1993; Saunders, Aasland, Babor, *et al.*, 1993; Allen *et al.*, 1997; Higgins-Biddle and Babor, 2018; Meneses-Gaya *et al.*, 2009; Reinert and Allen, 2002; Wardell *et al.*, 2020). Comparisons of scores with diagnostic data have allowed validation of the sub-division of AUDIT scores across 4 different tiers of alcohol use: low risk (0–7); hazardous (8–15); harmful (16–19) and severe dependence (≥20) (Conigrave *et al.*, 1995; Daeppen *et al.*, 2000; Babor *et al.*, 2001; Reinert and Allen, 2007). Research, within primary care settings, has predominately focused on employing the AUDIT (Fiellin *et al.*, 2000; Reinert and Allen, 2002; Meneses-Gaya *et al.*, 2009) and short forms such as the FAST (Hodgson *et al.*, 2002, 2003; Gómez *et al.*, 2005; Meneses-Gaya *et al.*, 2009, 2010; Jones, 2011) to distinguish between non-hazardous and hazardous alcohol use (i.e. as indicated by an AUDIT score ≥ 8, or a FAST score of ≥3). However, being able to quickly and accurately distinguish between the top 2 tiers of alcohol use (i.e. harmful and severe dependence) is crucial for appropriate signposting, especially within tertiary services such as specialist treatment centres (Babor and Robaina, 2016).

The ability to quickly and accurately place an individual’s alcohol usage within the appropriate tier is essential in the application of a stepped care model, in which individuals are paired with the least intensive level of intervention that fulfils their treatment needs (Sobell and Sobell, 1999, 2000). Such a stepped care model, which underpins many addiction treatment guidelines (Gastfriend and Mee-Lee, 2004; National Institute for Health and Care Excellence, 2011), allows individuals to be appropriately stepped up or down to the most suitable service, thereby maximizing cost-effectiveness and treatment efficacy (Sobell and Sobell, 1999, 2000; Jaehne *et al.,* 2012). The cost of intervention and treatment increases alongside the severity of alcohol use and misclassification into a higher treatment level can result in the unnecessary use of valuable resources (Sobell and Sobell, 1999, 2000). More importantly, however, misclassification of high-risk alcohol users into a lower treatment level deprives the individual from access to crucial and timely treatment (Drummond *et al.*, 2009; Jaehne *et al.*, 2012). As such, the validation of shorter, time-saving screening measures for the upper tiers of alcohol use is necessary to ensure appropriate signposting (Meneses-Gaya *et al.*, 2010), for those individuals with high alcohol consumption.

Given that the FAST has already demonstrated efficacy with regards to distinguishing between non-hazardous and hazardous alcohol use (Hodgson *et al.*, 2002, 2003; Gómez *et al.*, 2005; Meneses-Gaya *et al.*, 2009, 2010; Jones, 2011), the primary aim of the present research was to provide clarity as to the efficacy of the FAST, in comparison to the full AUDIT, as a BSI to identify harmful and dependent alcohol use. As a secondary aim, the present paper also examines the performance of the FAST against two other short forms of the AUDIT: the AUDIT-C and the AUDIT-3. While the AUDIT-C and the AUDIT-3 have shown some degree of efficacy for the identification of hazardous drinking in various settings (e.g. Bush *et al.*, 1998; Gordon *et al.*, 2001; Gual, 2002; Rumpf *et al.*, 2002; Gómez *et al.*, 2005), the full AUDIT appears to exhibit superior performance to both short forms (Gordon *et al.*, 2001; Kriston *et al.*, 2008). Both the AUDIT-C and the AUDIT-3 utilize solely consumption targeted questions from the AUDIT as a means of identifying alcohol consumption-related risk, whereas the FAST utilizes all indices, measuring not only alcohol consumption but also alcohol dependence and experience of alcohol-related harm. As such, it was predicted that the supplementary information collected by the FAST would allow for a more accurate distinction between the tiers of harmful and dependent alcohol use. The analysis of the data utilized in the present study was not pre-registered and the results should be considered exploratory.

## METHOD

### Design

The present paper employed a secondary data analysis which utilized non-parametric receiver operating characteristic (ROC) analyses in conjunction with measures of sensitivity and specificity.

### Participants

Data of 3366 individuals (age (years): *M* = 41.6, *SD* = 13.76, Range = 18–97; Female = 1,309) were compiled from full AUDIT datasets collected across a range of settings throughout South Wales, UK. Given that the primary aim of the research was to investigate the ability of the FAST in its identification of harmful and severely dependent levels of alcohol use, datasets were requested from populations where individuals displaying such levels of alcohol use were likely to be present. The datasets included data from A&E departments, community mental health and addictions teams, and a community sample from the general population, which was comprised of data collected from the support organization Alcohol Change and research on student populations. The University of South Wales requested data from each of these sources and was provided with authorized access to select demographic information, including gender and age, as well as itemized AUDIT scores. Only complete cases, which included the required demographic information of age and gender, were carried forward for analysis (see Table 1).

**Table 1:** Distribution of participants tested and those included in inferential analysis

		**Total**					**Inferential analysis**			
				**Age**					**Age**	
**Dataset**		*n*	Female	*M*	*SD*		*n*	Female	*M*	*SD*
**Combined**		3366	1309 (38.9%)	41.63	13.76		3,356	1302 (38.8%)	41.64	13.68
**CS**		288	158 (55.0%)	33.90	14.76		288	158 (55.0%)	33.90	14.76
**A&E**		679	318 (46.8%)	36.11	15.89		671	311 (46.3%)	36.09	15.92
**CMHAT**		2399	833 (34.7%)	44.09	12.02		2397	833 (34.8%)	44.13	11.96


**Note:** M = mean, SD = standard deviation, CS = Community Sample, A&E = Accident and Emergency, CMHT = community Mental Health & Addictions Teams.

### Materials and procedures

#### AUDIT

As part of routine data collection, individuals completed the AUDIT (Saunders and Babor, 1993). The AUDIT was developed by the World Health Organization and has been validated for use within numerous countries to help identify those at risk from alcohol misuse, including the UK (Cherpitel *et al.*, 2005; Meneses-Gaya *et al.*, 2009; Johnson *et al.*, 2013; Foxcroft *et al.*, 2014; Higgins-Biddle and Babor, 2018). The test is comprised of 10 items and utilizes a 5-point Likert scale from 0 to 4 to score each of the items. All questions are the same regardless of gender, with the exception of question 3 which examines the frequency of consumption of 6 or more units for females and 8 or more units for males. Questions examine alcohol intake and frequency of use (Qs. 1–3), as well as psycho-social aspects of alcohol use such as dependence upon alcohol (Qs. 4–6), and experience of alcohol-related harm (Qs. 7–10) (Babor *et al.*, 2001). Summation of scores from all items generates an AUDIT total and provides a continuous index of alcohol use severity (0–40). The AUDIT Manual (Babor *et al.*, 2001) suggests 4 tiers of AUDIT scores that correspond with different levels of alcohol use, each with associated intervention strategies (described in Table 2). It should be noted, however, that these are general guidelines and, dependent upon the setting and population studied, the cut-off points for each subdivision may vary (see Meneses-Gaya *et al.*, 2009). For example, it has been suggested that a cut-off score of 5 for women, rather than 8, would be more beneficial in distinguishing between non-hazardous from hazardous alcohol use (Reinert and Allen, 2002, 2007).

**Table 2:** Total AUDIT score subdivisions with associated levels of risk and suggested interventions

**Tier**	**AUDIT score**		**Risk level**		**Intervention**
**1**	0–7		Abstinence/non-hazardous use		Alcohol education
**2**	8–15		Medium risk/hazardous use		Simple advice & education
**3**	16–19		Harmful use/possible dependence		Simple advice, brief counselling & continued monitoring
**4**	≥20		Severe alcohol dependence		Specialist referral for diagnostic evaluation & treatment

#### FAST

The FAST (Hodgson *et al.*, 2002) is a BSI that was developed for use in busy medical settings such as A&E hospital departments. It is comprised of questions 3, 5, 8 and 10 from the AUDIT and, as such, contains questions that examine consumption, dependence and experience of alcohol-related harm. Scores are summated to give a total FAST score, between 0 and 16, that can be used as a quick indicator of alcohol use severity. The FAST has previously demonstrated suitable validity for distinguishing non-hazardous from hazardous drinkers (Hodgson *et al.*, 2002, 2003; Meneses-Gaya *et al.*, 2010; Jones, 2011). There are two methods of score calculation. The first method utilizes question 1 to identify individuals as either non-hazardous (≤2) or hazardous (≥3) drinkers. Where individuals are identified as hazardous drinkers by question 1, data collection is subsequently halted. The second method involves the summation of scores from all questions, regardless of the score from question 1, with scores of ≥3 indicating a hazardous level of alcohol use. The present study utilizes the latter method of FAST scoring.

#### AUDIT short forms

To provide a comparison with other short forms of the AUDIT, scores for the AUDIT-C and AUDIT-3 were also calculated. The AUDIT-C and the AUDIT-3 utilize the consumption targeted questions from the AUDIT as a means of identifying alcohol consumption-related risk, with both short forms having demonstrated some degree of efficacy for the identification of hazardous drinking in a variety of settings (e.g. Bush *et al.*, 1998; Gordon *et al.*, 2001; Gual, 2002; Rumpf *et al.*, 2002; Gómez *et al.*, 2005). The AUDIT-C summates answers from questions 1–3 of the AUDIT, whereas the AUDIT-3 utilizes only the third item from the AUDIT which assesses the frequency of heavy episodic drinking (Bush *et al.*, 1998).

### Analyses

**Figure 1.**
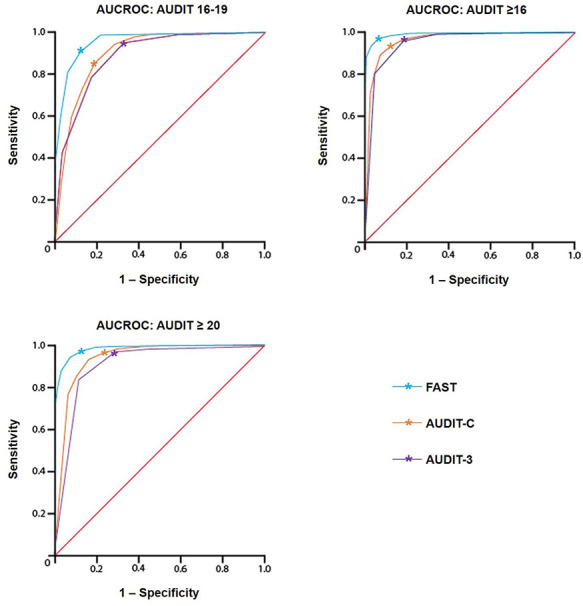
**ROC curve for the FAST, AUDIT-C and AUDIT-3, for AUDIT cut-offs for harmful and dependent alcohol use;** ^*^ denotes suggested cut-off scores for AUDIT short forms.

All data were analysed using SPSS statistical analyses software for Windows (Version 25.0. Armonk, NY: IBM Corp). To examine the accuracy with which total FAST scores were able to predict the subdivisions of harmful drinking and severe alcohol dependency (as identified by total AUDIT scores of 16–19 and ≥20, respectively), ROC analyses were conducted. Additionally, ROC analysis for harmful alcohol use was also calculated utilizing scores of ≥16 to demonstrate how utilizing such a method may influence results. Good accuracy was indicated by area under the ROC curve (AUROC) scores of ≥0.80, and excellent accuracy was indicated by AUROC scores of ≥0.90. Sensitivity (i.e. the probability that a FAST score will accurately predict inclusion within an AUDIT subdivision [True Positive]) and specificity (i.e. the probability that a FAST score will accurately predict exclusion from within an AUDIT subdivision [True Negative]) were calculated for each AUDIT/FAST cut-off point. Additionally, to provide a comparison of efficacy with other short forms of the AUDIT, AUROC scores, sensitivity and specificity were calculated for the AUDIT-C and AUDIT-3 (Table 4 and Figure 1).

## RESULTS

The sample was characterized by a high level of alcohol consumption with average AUDIT scores indicating a general level of severe alcohol dependence (see Table 3). Of those individuals included in the inferential analysis (Table 1), 240 (7.2%) of the sample had AUDIT scores of 16–19, classifying their alcohol use within the 3rd tiers of harmful alcohol use, and 1986 individuals (66%) of the sample had AUDIT scores of ≥20, classifying their alcohol use within the 4th tier and as severely dependent.

**Table 3:** Mean and standard deviation of scores^*^ for the AUDIT and AUDIT short forms

	**Total** (N = 3366)				**Inferential analysis** (*n* = 3356)		
**Measure**		M	SD			M	SD
**AUDIT**		20.94	12.48			20.98	12.47
**FAST**		8.20	5.36			8.20	5.35
**AUDIT-C**		8.93	4.01			8.94	1.50
**AUDIT-3**		2.85	1.50			2.86	4.01

**Note:** M = mean, SD = standard deviation, AUDIT = Alcohol Use Disorder Identification Test, ^*^ = collated datasets.

AUROC scores for the divisions of AUDIT scores of 16–19, ≥16 and ≥20 were higher for the FAST than for either the AUDIT-C or AUDIT-3, respectively (see Table 4 and Figure 1).

**Table 4:** AUROC scores, sensitivity and specificity for FAST, AUDIT-C and AUDIT-3 scores contrasted against AUDIT cut-offs

			**AUDIT 16–19 (Harmful)**						**AUDIT ≥ 16 (Harmful)**						**AUDIT ≥ 20 (Severe dependence)**					
			AUROC			Sensitivity	Specificity		AUROC			Sensitivity	Specificity		AUROC			Sensitivity	Specificity	
**Method**	**Score**		Score	LB	UB	(TP) %	(TN) %		Score	LB	UB	(TP) %	(TN) %		Score	LB	UB	(TP) %	(TN) %
**FAST**	≥4		0.885	0.867	0.903	98.8	78.3		0.890	0.876	0.905	99.8	78.3		0.824	0.807	0.840	99.9	64.8
	≥5		0.889^^*^^	**0.865**	**0.914**	**90.0**	**87.9**		0.932	0.921	0.944	98.6	87.9		0.869	0.855	0.884	99.6	74.2
	≥6		0.874	0.844	0.904	80.8	94.0		0.955^^*^^	**0.946**	**0.964**	**97.0**	**94.0**		0.899	0.887	0.912	99.0	80.9
	≥7		0.782	0.742	0.821	59.2	97.2		0.952	0.944	0.961	93.3	97.2		0.935^^*^^^^*^^	**0.913**	**0.935**	**97.4**	**87.3**
	≥8		0.681	0.638	0.725	36.7	99.6		0.938	0.929	0.946	88.0	99.6		0.923	0.927	0.947	94.2	93.2
	≥9		0.567	0.533	0.619	15.4	99.7		0.898	0.887	0.908	79.8	99.7		0.932	0.913	0.933	87.6	97.1
	≥10		0.513	0.472	0.553	2.5	100		0.857	0.845	0.869	71.4	100		0.897	0.885	0.908	79.8	99.6
																			
**AUDIT-C**	≥5		0.768	0.743	0.793	100	53.6		0.768	0.748	0.787	100	53.6		0.721	0.702	0.740	99.9	44.2
	≥6		0.805	0.781	0.828	98.3	62.6		0.812	0.794	0.830	99.7	62.6		0.759	0.741	0.777	99.9	51.9
	≥7		0.837	0.813	0.861	95.0	72.4		0.857	0.841	0.874	99.1	72.4		0.801	0.784	0.818	99.6	60.6
	≥8		0.837^^*^^	**0.808**	**0.866**	**85.8**	**81.6**		0.895	0.881	0.909	97.4	81.6		0.843	0.828	0.858	98.8	69.8
	≥9		0.806	0.771	0.841	72.9	88.3		0.911^^*^^	**0.899**	**0.924**	**93.9**	**88.3**		0.870^*^^*^	**0.856**	**0.884**	**96.5**	**77.6**
	≥10		0.764	0.725	0.803	59.6	93.2		0.914	0.903	0.925	89.6	93.2		0.886	0.873	0.899	79.8	99.6
	≥11		0.690	0.648	0.733	42.1	96.0		0.884	0.872	0.896				0.874	0.861	0.887	70.5	99.8
																			
**AUDIT-3**	≥1		0.703	0.674	0.732	91.2	41.4		0.704	0.683	0.724				0.669	0.649	0.688		
	≥2		0.810^^*^^	**0.785**	**0.836**	**95.0**	**67.1**		0.829	0.812	0.812	98.8	67.1		0.777	0.760	0.795	99.2	56.2
	≥3		.805	.773	.838	78.3	82.7		.891^^*^^	**.878**	**.878**	**95.6**	**82.7**		.848^*^^*^	**.833**	**.864**	**97.6**	**72.0**
	=4		0.693	0.650	0.736	42.5	96.1		0.879	0.867	0.867	79.8	96.1		0.868	0.855	0.881	84.3	89.3

**Note:** AUDIT = Alcohol Use Disorder Identification Test, AUROC = Area Under Receiver Operating Characteristic Curve, LB = 95% confidence interval: lower bound, UB = 95% confidence interval: upper bound, ^*^ = suggested cut off for harmful alcohol use, ^*^^*^ = suggested cut off for dependent alcohol use.

For reference, Table 5 provides a percentage comparison of the scores of each short form of the AUDIT, with the total potential score available for that method or assessment.

**Table 5:** Scores displayed as a percentage of the total potential available scores for each measure

		**% of PTS**			
**Score**		FAST (PTS = 16)	AUDIT-C (PTS = 12)	AUDIT-3 (PTS = 4)	
≥1		6.25	8.33	25.00	
≥2		12.50	16.67	50.00^^*^^	
≥3		18.75	25.00	75.00^*^^*^	
≥4		25.00	33.33	100.00	
≥5		31.25^^*^^	41.67		
≥6		37.50	50.00		
≥7		43.75^*^^*^	58.33		
≥8		50.00	66.67^^*^^		
≥9		56.25	75.00^*^^*^		
≥10		62.50	83.30		
≥11		68.75	91.63		

**Note:** AUDIT = Alcohol Use Disorder Identification Test, ^*^ = suggested cut off for harmful alcohol use, ^*^^*^ = suggested cut off for dependent alcohol use. Full AUDIT Tier 3 cut off (≥16 = 40% PTS). Full AUDIT Tier 4 cut off (≥20 = 50% PTS).

## DISCUSSION

The FAST was initially developed for use as a BSI to identify hazardous alcohol use within busy medical care settings (Hodgson *et al.*, 2002) and has subsequently been utilized in number of settings (Meneses-Gaya *et al.*, 2010; Jones, 2011; Coulton *et al.*, 2012; Hallingberg *et al.*, 2015; Teferra *et al.*, 2016). The present paper aimed to investigate the efficacy of the FAST as a time- and cost-effective method of identifying harmful and dependent alcohol use, within a population which predominantly exhibits risk-related alcohol usage (66% were classed as severely dependent according to full AUDIT scores), as determined by the guidelines suggested within the AUDIT Manual: 2nd Edition (Babor *et al.*, 2001). The FAST is derived from the AUDIT and utilizes four questions from the 10-question full version that measure alcohol consumption, alcohol dependence and experience of alcohol-related harm. Therefore, as a secondary aim, we compared the efficacy of the FAST for identifying harmful and dependent alcohol use with two other BSI’s derived from the AUDIT: the AUDIT-C and the AUDIT-3. Crucially, these other short forms utilized solely consumption targeted questions from the AUDIT. Using AUROC analysis, we demonstrated that the FAST was able to accurately identify both harmful and dependent alcohol use, exhibiting superior performance to both the AUDIT-C and the AUDIT-3. The implications of our findings, and suggested FAST cut-offs for tiers of alcohol use, are discussed below.

The AUDIT, from which the FAST is derived, has long been used to provide a measure of an individual’s alcohol consumption, with AUDIT scores sub-divided into 4 tiers of alcohol dependence (non-hazardous; hazardous: harmful: severe dependence), each with an associated form of suggested treatment or intervention (Babor *et al.*, 2001). The AUDIT takes ~2 minutes to complete (Babor *et al.*, 2001) which, within busy health care or support settings, poses a limitation to its regular implementation. Consequently, shorter forms of the AUDIT have been developed as BSIs to help systematically identify an individuals’ level of alcohol use, in a more time-effective manner, before signposting the individual in question to the appropriate treatment intervention and/or for formal assessment, accordingly. By comparison, the FAST, which has been suggested for use in settings where time is limited (National Institute for Health and Care Excellence, 2020), takes ~45 seconds to complete (Hodgson *et al.*, 2002, 2003). However, as with the full AUDIT, research into the shorter forms has largely been limited to distinguishing between non-hazardous alcohol use (Tier I) and hazardous alcohol use, i.e. alcohol use that demonstrates any level of risk (Tiers >1) (Wardell *et al.*, 2020).

The FAST has previously demonstrated validity for distinguishing non-hazardous from hazardous alcohol use (Hodgson *et al.*, 2002, 2003; Meneses-Gaya *et al.*, 2010; Jones, 2011), with a score of ≥3 indicating a hazardous level of alcohol use. However, there are profound health and social implications for not correctly identifying harmful or dependent alcohol use, and so not implementing the appropriate intervention (Schuckit and Smith, 2000; Leonard and Eiden, 2007; Lönnroth *et al.*, 2008; Erol and Karpyak, 2015). Erring on the side of caution, sensitivity may be considered the most important characteristic when screening for problematic alcohol use (Knight *et al.*, 2003) as the potential cost:benefit ratio for identification as a false positive needs to weighed against that for identification of an individual as a false negative. In the case of a false positive, where an individual is incorrectly identified as needing a treatment or intervention that exceeds their alcohol use, they will either will receive or refuse the suggested intervention and the ramification of incorrect classification is largely financial. However, in the case of a false negative, where individuals with more problematic alcohol use are signposted to an insufficient treatment or intervention, the potential ramifications are much greater and include both societal and health implications. In the application of a stepped care model, as utilized by many health care and treatment services, it is therefore essential to be able to quickly and accurately identify an individual’s alcohol usage and pair it with the most appropriate level of treatment or intervention, to maximize cost-effectiveness and treatment efficacy (Sobell and Sobell, 1999, 2000). The findings of the present research demonstrate the ability of the FAST to successfully identify both harmful and dependent alcohol use, extending its usefulness as a BSI beyond distinguishing between non-hazardous or hazardous alcohol use. It is the suggestion of the authors that a FAST score of ≥5 is utilized for the identification of harmful alcohol use, and a FAST score of ≥7 is utilized for the identification of dependent alcohol use. These findings are in line with prior research which has typically used a cut-off of ≥3 to identify hazardous drinking (e.g. Meneses-Gaya *et al.*, 2010; Jones, 2011; Coulton *et al.*, 2012; Hallingberg *et al.*, 2015; Teferra *et al.*, 2016). When contrasted against AUDIT scores (16–19) for the harmful tier of alcohol use, a FAST score of ≥5 displayed an associated AUCROC score of 0.889, with sensitivity and specificity values of 90 and 87.9%, respectively. It should be noted that results based on calculations that classified harmful use as ≥16 produced an optimum AUCROC score of .955 for a FAST score of ≥6, with associated sensitivity and specificity values of 97 and 94%, respectively. The contrast between the two methods serves to highlight how, when calculating the cut-offs for tiers of alcohol use for BSIs, the inclusion of data from individuals in higher tiers may have detrimental consequences, with individuals being subsequently referred to a lower treatment intervention than that which they may require. When contrasted against AUDIT scores (≥20) for the dependent tier of alcohol use, a FAST score of ≥7 had an associated AUCROC score of 0.935 with sensitivity and specificity values of 97.4 and 87.3%, respectively. When contrasted against the AUDIT-C and the AUDIT-3, the FAST exhibits superior performance as a BSI for correctly identifying individuals at risk of harmful and dependent alcohol usage. In comparison to the FAST, both the AUDIT-C and AUDIT-3 had lower associated AUCROC scores for harmful (AUDIT: 16–19) and dependent (AUDIT: ≥20) alcohol use. Notably, both the AUDIT-C and AUDIT-3 had comparable sensitivity to the FAST but displayed much lower specificity. Additionally, the highest AUCROC scores for both the AUDIT-C and AUDIT-3 were achieved at a much higher percentage of the potential total scores (PTS) than for the FAST. Scores of 5 and 7 of the FAST equate to a 31.25 and 43.75% of the PTS, which is comparable to the AUDIT scores of 16 and 20 for harmful and dependent alcohol use, which equate to 40 and 50% of the PTS, respectively. In contrast, the highest AUCROC scores for harmful and dependent alcohol use, for the AUDIT-C, were achieved at 66.67 and 75% of the PTS, respectively; whereas the scores for the AUDIT-3 were achieved at 50 and 75% of the PTS. Therefore, for the consumption only short-forms of the AUDIT, a higher rate of alcohol consumption would be required for the identification of harmful or severely dependent alcohol use, potentially risking the well-being of those individuals who do not meet the threshold but would otherwise have been identified using the FAST. The inclusion of questions in the FAST, beyond those relating to consumption (e.g. level of harm and dependence), is consistent with the full AUDIT in that questions related to impaired control over drinking and alcohol-related harm provide supplementary information which serves to indicate the need for a full diagnostic assessment by a trained clinician and/or referral (Babor *et al.*, 2001; Higgins-Biddle and Babor, 2018). This inclusion of such indices allows for a more efficacious means of determining inclusion within the tiers of alcohol use set out for the AUDIT. For example, some individuals may be more susceptible to alcohol-related harm than others, due to factors that impact upon their tolerance for alcohol consumption, e.g. age, gender or physiology (Higgins-Biddle and Babor, 2018). In short, when contrasted against the AUDIT-C and the AUDIT-3, the FAST exhibits superior performance as a BSI for correctly identifying individuals at risk of problematic alcohol usage, providing a more efficacious means for signposting to the appropriate intervention and reducing the potentially for inappropriate allocation of resources.

### Limitations and future considerations

The data sets, which were analysed in the present research, were chosen because they were likely to yield a high proportion of individuals with levels of alcohol usage which would be considered risk-related (i.e. predominantly within the tiers of harmful and severely dependent). Many individuals who fall within the tiers of harmful and severely dependent alcohol usage are likely to be assessed within settings such as Accident & Emergency departments or community mental health and addictions services. The present research, therefore, provides a good indication of the applicability of the FAST as a BSI within such populations, and it would be expected that future research may observe comparable sensitivity and specificity when examining FAST, AUDIT-3 or AUDIT-C in similar populations. However, the authors acknowledge that the high prevalence of risk-related alcohol usage observed in the present study may influence the predictive strengths of the tests examined.

Sensitivity and specificity are intrinsic properties of a measurement tool and are typically considered robust to variations of frequency or prevalence (Linden, 2006; de Torres *et al.*, 2009). However, a number of studies indicate that variations in disease prevalence may exert more of an influence over sensitivity and specificity than previously thought (Brenner and Gefeller, 1997; Mulherin and Miller, 2002; Leeflang *et al.*, 2009), most likely via mechanisms which impact upon clinical variability and patient spectrum (e.g. variations in the distribution of symptoms and their severity) (Leeflang *et al.*, 2013). The influence that prevalence may play upon classification within a spectrum of condition severity, as opposed to a binary classification (i.e. the patient either has or does not have a disease), is unclear. However, it cannot be ruled out that large variation in alcohol usage, between populations, may contribute to observed differences in the predictive values obtained. The degree of alcohol usage within the general population and other clinical and/or community settings will likely differ and may well be associated with additional population characteristic differences (Lundin *et al.*, 2015). Therefore, replication studies using a more diverse range of participants are required to ensure that the validity of these measures as BSIs is not overstated (de Torres *et al.*, 2009; Meneses-Gaya *et al.*, 2009). Consequently, the findings contained within the present research do not necessarily indicate that a comparable level of efficacy for the FAST as a BSI, with regards to the accurate identification of the upper tiers of alcohol usage, would be observed within different sample populations and cut-off points should therefore be calculated separately for each target setting utilizing samples similar to the targeted population. Additionally, our findings cannot be generalized to underage/adolescent drinkers (≤17 years of age) as they were not included in our sample. Future studies may also wish to address this limitation by examining the applicability of the FAST for all tiers of alcohol usage in populations of alcohol users who are under the legal drinking age.

## CONCLUSION

To the best of our knowledge, we are the first to examine the efficacy of three short forms of the AUDIT, the FAST, the AUDIT-C and the AUDIT-3, with regards to the identification of the upper tiers of alcohol usage, as defined by the AUDIT. The FAST demonstrates efficacy as a BSI, in the correct identification of harmful and dependent levels of alcohol use. Building upon the ability of the FAST to successfully distinguish between hazardous and non-hazardous alcohol use, the present paper demonstrates its potential for use as a cost- and time-effective method for appropriate screening and signposting in the stepped care model utilized by many health care and treatment services. Notably, in comparison to two other short forms of the AUDIT (AUDIT-C and the AUDIT-3), the FAST exhibits superior performance as a BSI, with regards to its ability to successfully distinguish between the upper tiers of alcohol use. These findings provide additional evidence to indicate that screening for alcohol consumption alone is not sufficient to effectively distinguish between alcohol usage that falls within the tiers of harmful and severely dependent levels of alcohol use. Future investigations should consider the need, within the field of addictions research, for robust replication of studies, using large sample sizes and a variety of populations (Heirene, 2020).

## Data availability statement

The data underlying this article will be shared on reasonable request to the corresponding author.

## Conflict of interest statement

All authors declare no conflict of interest.
